# Structural Analysis of Metabolites of Asiatic Acid and Its Analogue Madecassic Acid in Zebrafish Using LC/IT-MS^n^

**DOI:** 10.3390/molecules20023001

**Published:** 2015-02-12

**Authors:** Binbin Xia, Lu Bai, Xiaorong Li, Jie Xiong, Pinxiang Xu, Ming Xue

**Affiliations:** Beijing Laboratory for Biomedical Detection Technology and Instrument, Department of Pharmacology, School of Basic Medical Sciences, Capital Medical University, Beijing 100069, China; E-Mails: xiabinbin2006@163.com (B.X.); lubai2009@163.com (L.B.); madingdaier@aliyun.com (X.L.); xiongjie22@hotmail.com (J.X.); syxpx88@163.com (P.X.)

**Keywords:** zebrafish, metabolism, asiatic acid, madecassic acid, LC/IT-MS^n^

## Abstract

Although zebrafish has become a significant animal model for drug discovery and screening, drug metabolism in zebrafish remains largely unknown. Asiatic acid (AA) and madecassic acid (MA), two natural pentacyclic triterpenoids mainly obtained from *Centella asiatica* (L.) Urban, have been found to possess many pharmacological effects. This study is to probe the metabolic capability of zebrafish via investigation of the drug metabolism of AA and MA in zebrafish, using a sensitive LC/IT-MS^n^ method. In addition, the main fragmentation pathways of AA and MA were reported for the first time. Nineteen metabolites of AA and MA were firstly identified after zebrafish was exposed to the drug, which all were the phase I metabolites and mainly formed from hydroxylation, dehydrogenation, hydroxylation and dehydrogenation, dihydroxylation and dehydrogenation, and dehydroxylation reaction. The results indicated that zebrafish possessed strong metabolic capacity, and the metabolites of AA and MA were formed via similar metabolic pathways and well matched with the known metabolic rules *in vivo* and *in vitro*, which supports the widely use of this system in drug metabolism research. This investigation would also contribute to the novel information on the structural elucidation, *in vivo* metabolites and metabolic mechanism of pentacyclic triterpenoids.

## 1. Introduction

Identification of the *in vivo* metabolites of drug candidates provides very important information for evaluating the efficacy, toxicity and stability, resulting in discovery and development of novel drugs. It is very significant and necessary to study the metabolites and metabolic pathways for the candidates in the early stage of drug development. *Centella asiatica* (L.) Urban is a well-known traditional Chinese medicine (TCM) used in treatment of many diseases such as jaundice, heatstroke, diarrhea, skin disease, hepatitis and cerebrospinal meningitis in clinical practice [1]. Asiatic acid (AA) and its analogue madecassic acid (MA) are the major active components that isolated and identified from *Centella asiatica* (L.) Urban, which are the aglycones of ursane-type pentacyclic triterpenoids. Recent studies demonstrated that AA and MA had significant effects in treatment of skin wound [2,3], inflammation [4,5,6,7], anti-oxidant [8,9,10], tumor [11,12,13] and nerve damage [14,15,16]. Despite that the pharmacological activities of AA and MA have been well reported, the mass spectral fragmentation pattern, *in vivo* metabolites, metabolic pathways and characteristics of AA and MA, especially in the mode animal zebrafish, are absent. 

Zebrafish is increasingly used in drug screening and toxicological studies owing to the high-throughput advantages [17]. Since the developmental and physiological processes are highly conserved between zebrafish and mammals, drugs designed to interact with complicated processes of interest in zebrafish usually produce a similar pharmacological effects in human and mammals [18]. Especially, a variety of metabolic enzymes such as cytochrome P_450_ family, epoxide hydrolase, and conjugation enzymes, are markedly expressed in zebrafish [19,20,21,22,23,24] that are suitable for studying drug metabolism with obvious advantages of low cost and high efficiency.

In order to confirm the metabolic capacity of zebrafish and better understand the mass spectral fragmentation pattern, *in vivo* metabolic pathways and characterization of AA and MA, a novel and sensitive LC/IT-MS^n^ method was established to study the MS structural characteristics of the metabolites of AA and MA in zebrafish. All of these findings contributed a further understanding of the intermediate processes and metabolism mechanism of these pentacyclic triterpenoids. The *in vivo* metabolism study of AA and MA might also provide useful and important information for the further studies pharmacological activity of these compounds. Our investigation has provided much novel information on *in vivo* metabolism of pentacyclic triterpenoids, which would help novel drug development, as well as a better understanding of the safety and efficacy of these compounds.

## 2. Results and Discussion

### 2.1. Fragmentation Pathways of AA and MA

This investigation involved the chromatographic and mass spectral properties of the parent drug. The chromatographic and mass spectrometry conditions were optimized for maximum abundances of the ions of the interests by the automatic tune procedure of the instrument. The first step in our work involved the characterization of chromatographic and mass spectral properties of AA and MA, full scan mass spectral analyses for these two parents showed the deprotonated molecule ions of *m*/*z* 487 and 503 from LC/IT-MS^n^. To ensure sufficient fragment ions, 10 μg/mL of AA or MA prepared in methanol was used for the fragmentation pattern study. The data of MS^n^ spectra of AA and MA are shown in Table 1.

**Table 1:** Chromatographic retention times, mass spectrometric data of AA and MA.

Metabolites	Precursor Ion ([M−H]^−^)	Retention Time (min)	Data-Dependent MS^n^ Data (Collision Energy: 36%; Relative Abundance: % Base Peak)
AA	487	37.57	MS^2^[487]:487(35), 473(4), 443(3), 441(7), 423(4), 421(19), **409**(100), 393(8), 391(10), 379(11), 153(<2)
			MS^3^[487→441]:421(20), **409**(100), 379(15), 233(8)
			MS^3^[487→409]:391(50), **379**(100), 375(10)
			MS^3^[487→391]:**391**(100), 375(73), 373(49), 347(40), 189(48)
			MS^3^[487→379]:379(8), 377(43), **363**(100), 347(21), 225(3), 189(7), 175(13)
			MS^4^[487→441→409]:391(28), **379**(100), 226(36)
			MS^4^[487→409→379]:377(16), **363**(100), 361(60), 347(10), 225(14), 175(5)
MA	503	30.51	MS^2^[503]:503(96), 485(43), 465(12), 453(41), 435(33), 419(42), **407**(100), 391(37), 389(97), 373(19), 371(33), 363(7), 247(2), 203(17),201(3)
			MS^3^[503→485]:485(2), 465(14), 453(59), **435**(100), 409(64), 391(2), 201(2)
			MS^3^[503→407]:407(3), 405(20), **389**(100), 371(12), 159(3)
			MS^3^[503→389]:389(85), 387(18), **373**(100), 371(26), 361(44), 359(79), 201(36), 187(82)
			MS^4^[503→407→389]:389(2), **373**(100)
			MS^4^[503→485→453]:453(60), 451(35), **435**(100), 417(5), 391(2), 379(4), 201(10)
			MS^4^[503→485→435]:435(60), **407**(100), 391(14), 201(4), 173(26)
			MS^5^[503→485→453→435]:435, 417, 407, 391
			MS^3^[503→391]:**391**(100), 375(60), 363(33), 361(53), 359(3), 189(34), 187(4), 173(12)
			MS^4^[503→391→375]:**375**(100), 359(38), 345(2), 189(2), 173(<2)
			MS^4^[503→391→363]:**363**(100), 347(30), 223(<2), 173(30), 159(10)

**Figure 1:**
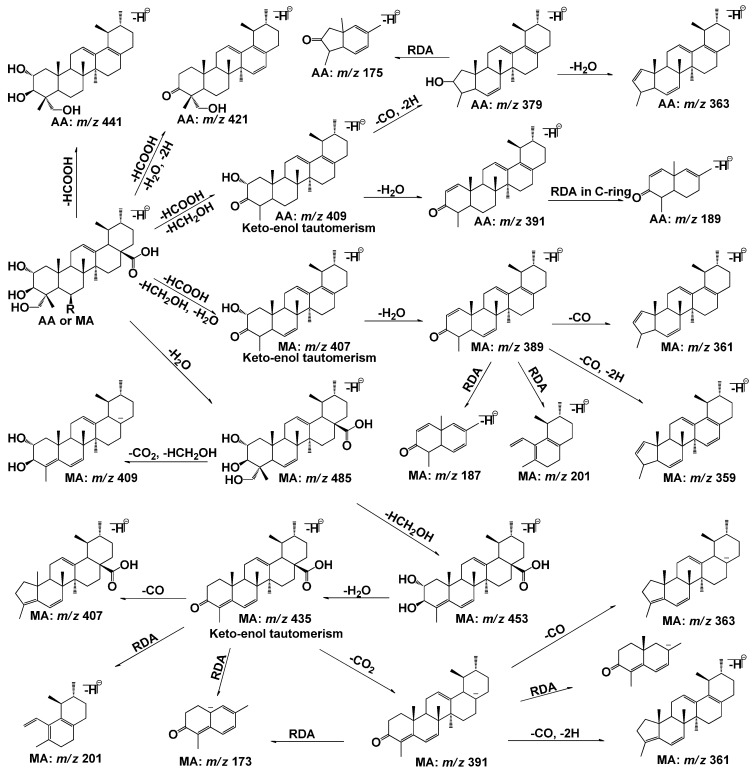
Main fragment ions and fragmentation pathways of the [M−H]^−^ ions of AA and MA (AA: R = H, MA: R = OH).

The [M−H]^−^ ions at *m*/*z* 487 and *m*/*z* 503 were observed as the quasi-molecular ion of AA and MA, respectively. The MS/MS spectrum of the [M−H]^−^ ion of AA exhibited a neutral loss of carboxyl and hydroxylmethyl group, leading to form the base peak ion at *m*/*z* 409 [25]. The MS/MS spectrum of the [M−H]^−^ ion of MA exhibited the neutral losses of HCOOH, HCH_2_OH, and H_2_O, leading to the formation of the base peak ion at *m*/*z* 407. The main fragment ions and fragmentation pathways of AA and MA were illustrated in Figure 1. Based on the above mass spectrum profiles of AA and MA, the neutral losses of CO_2_ (44 Da), HCOOH (46 Da), HCH_2_OH (32 Da), H_2_O (18 Da) and CO (28 Da) [26] occurred at these two compounds, and the retro-Diels–Alder (RDA) cleavage were regarded to be the characteristic fragmentation pathways for AA and MA or their analogues [26,27]. The findings help to identify and elucidate the *in vivo* metabolites of AA and MA.

### 2.2. Identification of AA and Its Metabolites with Zebrafish Exposure

**Figure 2:**
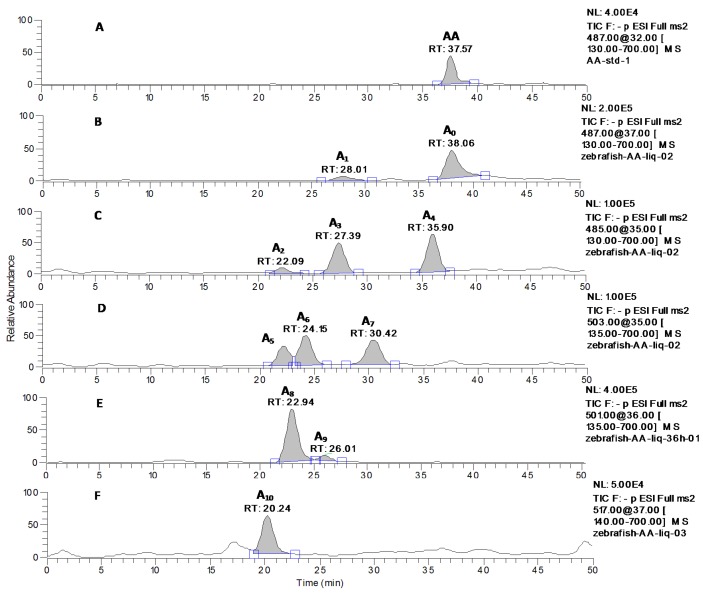
LC-MS/MS chromatograms of AA at *m*/*z* 487 (**A**) and its metabolites with zebrafish exposure at *m*/*z* 487 (**B**), *m*/*z* 485(**C**), *m*/*z* 503 (**D**), *m*/*z* 501(**E**), *m*/*z* 517(**F**).

**Figure 3:**
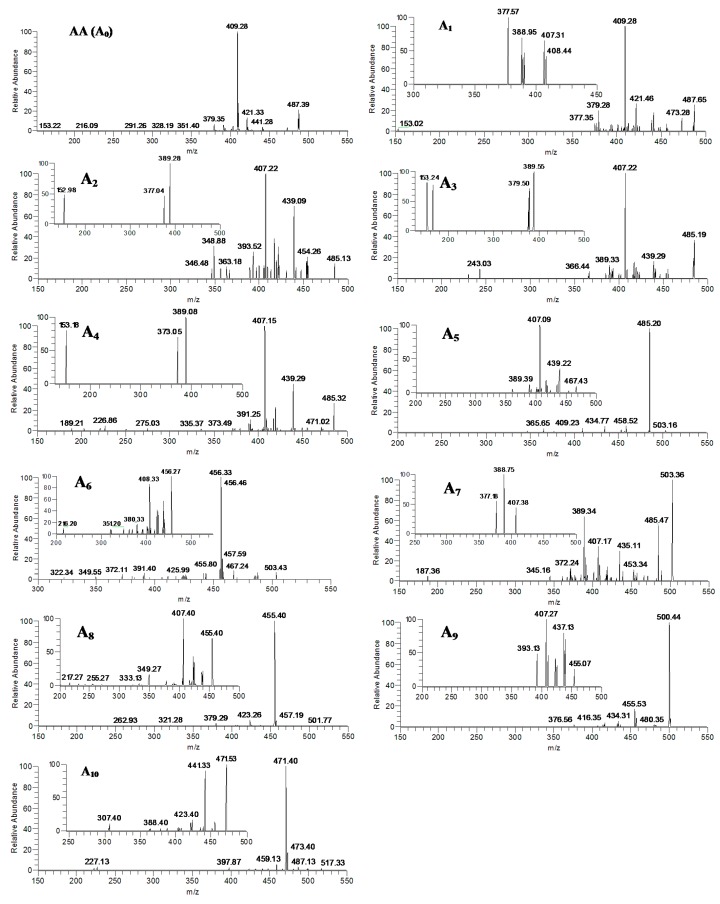
The MS^n^ (*n* = 2–3) spectra of the metabolites (A_0_–A_10_) of asiatic acid after zebrafish exposure: A_0_ (*m*/*z* 487); A_1_ (*m*/*z* 487, *m*/*z* 487→409), A_2_, A_3_ and A_4_ (*m*/*z* 485, *m*/*z* 485→407 ); A_5_ (*m*/*z* 503, *m*/*z* 503→485); A_6_ (*m*/*z* 503, *m*/*z* 503→457); A_7_ (*m*/*z* 503, *m*/*z* 503→407); A_8_ and A_9_ (*m*/*z* 501, *m*/*z* 501→455); and A_10_ (*m*/*z* 517, *m*/*z* 501→471).

AA was observed as the deprotonated molecule [M−H]^−^ at *m*/*z* 487 with the retention time of 37.57 min (Figure 2A). The retention time and the MS^n^ spectra of A_0_ were the same as those of the standard compound AA, indicating that A_0_ was the unchanged parent drug (Figure 2B and Figure 3). A_1_ was eluted at 28.01 min (Figure 2B) and gave rise to the quasi-molecular ion at *m*/*z* 487 ([M−H]^−^). The MS/MS spectra of A_1_ were similar to that of AA, and the main fragment ions were also produced by the same neutral loss from AA (Figure 3), forming the characteristic product ions at the *m*/*z* 441 ([m-HCOOH]^−^), *m*/*z* 421 ([m-HCOOH-H_2_O-2H]^−^), *m*/*z* 409 ([m-HCOOH-HCH_2_OH]^−^) and *m*/*z* 379 ([m-HCOOH-HCH_2_OH-CO-2H]^−^) (here, m = M-H). Thus, A_1_ was identified as the regio-isomer of AA. The data from the MS^3^ spectra of A_1_ (*m*/*z* 487→409) could further confirm this conclusion (Figure 3, Table 2).

The metabolite A_2_, A_3_ and A_4_ was detected at 22.09, 27.39 and 35.90 min, respectively (seen in Figure 2C) and gave rise to the same deprotonated quasi-molecular ions at *m*/*z* 485. All of these metabolites were 2 Da less than that of AA. These three metabolites exhibited almost identical mass spectral profiles, indicating that they were the isomers. The main fragment ions from the mass spectra of A_2_, A_3_ and A_4_ were also 2 Da less than those of AA (Figure 3). The mass spectra of the deprotonated ions of A_2_, A_3_ and A_4_ formed two predominated product ions at *m*/*z* 439 and 407 (see Figure 3), which were consistent with the sequentially neutral losses of HCOOH and HCH_2_OH. As a consequence, A_2_, A_3_ and A_4_ were identified as the isomers of the dehydrogenation products of AA. The data from the MS^n^ spectra (*m*/*z* 485→455, *m*/*z* 485→439, *m*/*z* 485→407 and *m*/*z* 485→407→389) of A_2_, A_3_ and A_4_ could further confirm this conclusion (shown in Figure 3 and Table 2).

The metabolite A_5_, A_6_ and A_7_, eluted at 22.22, 24.15 and 30.42 min, respectively (Figure 2D), were characterized with the quasi-molecular ion at *m*/*z* 503 ([M−H]^−^) and 16 Da over the parent drug (shown in Figure 3). The major product ion at *m*/*z* 485 was observed in the mass spectra of A_5_, corresponding to the cleavage of a H_2_O. The MS^3^ spectra of A_5_ (*m*/*z* 503→485), which was similar to the mass spectra of A_4_, formed main the fragment ions at *m*/*z* 439 ([m-HCOOH]^−^), *m*/*z* 407 ([m-HCOOH-HCH_2_OH]^−^) and *m*/*z* 389 ([m-H_2_O-HCOOH-HCH_2_OH]^−^) (here, m = M-H-H_2_O). The main product ions at *m*/*z* 457 and 456 were detected in the MS/MS spectra of A_6_ (see Figure 3), which was consistent with the neutral losses of a COOH group and a HCOOH group. The MS/MS spectra of A_7_ formed the main characteristic fragment ions at the *m*/*z* 485 ([m-H_2_O]^−^), *m*/*z* 453 ([m-H_2_O-HCH_2_OH]^−^), *m*/*z* 435 ([m-2H_2_O-HCH_2_OH]^−^), *m*/*z* 407 ([m-H_2_O-HCOOH-HCH_2_OH]^−^), and *m*/*z* 389 ([m-2H_2_O-HCOOH-HCH_2_OH]^−^), which was actually identical to the fragment ions of MA (here, m = M–H). The predominated characteristic product ion at *m*/*z* 389 was also observed in the MS^3^ spectra of A_7_ (*m*/*z* 503→407). Based the MS^n^ spectra of A_5_, A_6_ and A_7_ (shown in Table 2) and the data with Table 3, they were identified as the isomers of hydroxylation products of AA.

**Table 2:** Chromatographic retention times, mass spectrometric data of potential metabolites of AA (A_1_–A_10_) and MA (M_1_–M_9_).

Metabolites	Precursor Ion ([M−H]^−^)	Retention Time (min)	Data-Dependent MS^n^ Data (Relative Abundance: % Base Peak)
A_1_	487	28.01	MS^2^[487]: 487, 473, 443, 441, 421, **409(100)**, 379, 377, *etc.*
			MS^3^[487→409]: 407, 391, 389, **377(100)**
A_2_	485	22.09	MS^2^[485]: 485, 455, 439, 419, 417, **407 (100)**, 393 *etc.*
			MS^3^[485→455]: 455, 439, 425, **411 (100)**, 379
			MS^3^[485→439]: 439, 419, 417, **407 (100)**, 402, 393
			MS^3^[485→407]: **389(100)**, 377, 153
			MS^4^[485→407→389]: **389(100)**, 374,
A_3_	485	27.39	MS^2^[485]: 485, 455, 439, 419, 417, **407 (100)**, 391, 389, *etc.*
			MS^3^[485→455]: **411 (100)**, 391
			MS^3^[485→439]: 439, **407 (100)**, 371, 257
			MS^3^[485→407]: 389**(100)**, 379, 165, 153
			MS^4^[485→407→389]: 389, **371(100)**
A_4_	485	35.90	MS^2^[485]: 485, 471, 455, 439, 419, 417, **407 (100)**, 391, 389, 373, 189 *etc.*
			MS^3^[485→455]: **455(100)**, 407, 391, 389
			MS^3^[485→439]: **439(100)**, 419, 417, 407 (100)
			MS^3^[485→407]: **389(100)**, 373, 153
			MS^4^[485→407→389]: **389(100)**
A_5_	503	22.22	MS^2^[503]: 503, **485(100)**, 459, 435, 409, *etc.*
			MS^3^[503→485]: 467, 439, 419, 417, **407(100)**, 389
			MS^4^[503→485→407]: 391, 389, **377(100)**
A_6_	503	24.15	MS^2^[503]: 503, 487, 467, 457, **456(100)**, 443, 425, 411, 391, *etc.*
			MS^3^[503→457]: **456(100)**, 438, 424, 408, 392, *etc.*
			MS^4^[503→457→407]: **408(100)**, 407, 392, 390, 379, 377
A_7_	503	30.42	MS^2^[503]: **503(100)**, 485, 453, 435, 407, 391, 389, *etc.*
			MS^3^[503→453]: 453, 435, **423(100)**
			MS^3^[503→407]: 407, **389(100)**, 377
A_8_	501	22.94	MS^2^[501]: 501, 457, **455(100)**, 423, 379, *etc.*
			MS^3^[501→457]: 457, 455, 439, 437, **425(100)**, 407, 393, *etc.*
			MS^3^[501→455]: 455,439, 437, 425, 423, **407(100)**, 379, 349
			MS^3^[501→423]: **423(100)**, 407, 405, 389, 379
			MS^3^[501→379]: **379(100)**, 361, 353, 335
			MS^4^[501→455→407]: **407(100)**, 391, 379, 377, *etc.*
A_9_	501	26.01	MS^2^[501]: **501(100)**, 457, 455, 434, 416, 377 *etc.*
			MS^3^[501→455]: 455,439, 437, 425, 423, 409, **407(100)**, 393
A_10_	517	20.24	MS^2^[517]: 517, 499, 487, 473, **471(100)**, 459, *etc.*
			MS^3^[517→471]: **471(100)**, 455, 441, 423, 421, *etc.*
			MS^4^[517→471→441]: **441(100)**, 423, 409, 393, 391, 379, 375, 363, *etc.*
			MS^4^[517→471→455]: **455(100)**, 439, 407, 405
M_1_	501	18.50	MS^2^[501]: 501, **483(100)**, 457, 435, 419, 417, 407, 405, 389, 373, 361, *etc.*
			MS^3^[501→483]: 435, **407(100)**, 389, 371
			MS^3^[501→435]: **417(100)**, 389
			MS^3^[501→407]: **389(100)**, 371
			MS^3^[501→389]: **389(100)**, 373, *etc.*
M_2_	501	24.49	MS^2^[501]: 501, 483, 471, **455(100)**, 425, 407, 379, *etc.*
			MS^3^[501→455]: **455(100)**, 439, 437, 425, 423, 407, 389, 379, 361, *etc.*
			MS^3^[501→407]: 407, **389(100)**, 377
M_3_	501	28.86	MS^2^[501]: 501, **483(100)**, 457, 455, 437, 435, 419, 407,405, 389, 371, *etc.*
			MS^3^[501→455]: 483, 453, 437, 435, 417, 407, 405, 389, 387, **363(100)**
M_4_	501	36.40	MS^2^[501]: 501, 483, 463, 437, 435, 433, 419, 407,405, **389(100)**, 371, *etc.*
			MS^3^[501→389]: 389, **387(100)**
M_5_	487	32.51	MS^2^[487]: 487, 469, 457,441, **423(100),** *etc.*
			MS^3^[487→423]: **393(100)**, 467, 349
M_6_	487	37.93	MS^2^[487]: 487, 457,441, 419, **409(100)**, *etc.*
			MS^3^[487→409]: **379(100)**, 363
M_7_	517	17.12	MS^2^[517]: 517, 501, **471(100)**, 453, 423, 407, 405, 391, *etc.*
			MS^3^[517→471]: 453, 435, 423, **405(100)**, 387, 371, *etc.*
			MS^3^[517→453]: 435, **423(100)**, 405, 387, 347, 173
			MS^3^[517→423]: 407, **405(100)**, 389, 387, 357, 341
			MS^4^[517→471→453]: 453, 435, 425, 423, 407, **405(100)**, 389,387
			MS^4^[517→471→423]: 423, **405(100)**, 387, 357
			MS^4^[517→471→405]: **405(100)**, 387, 377, 371, 357, *etc.*
M_8_	519	13.47	MS^2^[519]: 519, **501(100)**, 483, *etc.*
			MS^3^[519→501]: 483, **435(100)**, 425
			MS^3^[519→483]: 483, 471, 435, 417, 407, 405, 389, 371, 363, 361, *etc.*
			MS^4^[519→501→483]: 465, **435(100)**, 407
M_9_	533	14.74	MS^2^[533]: 533, 489, **487(100)**, 469, 425, 403, *etc.*
			MS^3^[533→489]: 489, 471, 441, **423(100)**, 405, 381, 365, *etc.*
			MS^3^[533→487]: **487(100)**, 469, 457, 439, 421, 403, 363, *etc.*
			MS^4^[533→489→471]:423, 405, 395, 381, **363(100)**
			MS^4^[533→487→469]: 469, 454, **439(100)**, 421, 403, 387, 379

A_8_ and A_9_ were detected at 22.94 and 26.01 min, respectively (Figure 2E). Both of A_8_ and A_9_ shared the same deprotonated quasi-molecular ions at *m*/*z* 501([M−H]^−^), and were 14 Da more than that of AA. The characteristic fragment ion at *m*/*z* 455 was observed in the MS/MS spectra of A_8_ and A_9_ (Figure 3), which was consistent with the neutral loss of a HCOOH (46 Da). The MS^3^ spectra of A_8_ and A_9_ (*m*/*z* 501→455) that exhibited similar mass spectral profiles (seen in Figure 3), forming the main product ions at *m*/*z* 439, 437, 425, 423 and 407, could further confirm the results Base on the fragmentation pathways of AA, MA (Figure 1) and the data with Table 3, A_8_ and A_9_ were tentatively identified as the isomers of hydroxylation and dehydrogenation products, or the methylene to ketone product of AA.

A_10_, eluted at 20.24 min, gave rise to the deprotonated ion at *m*/*z* 517 (see Figure 2F). The major fragment ion at *m*/*z* 471 (Figure 3) was detected in the MS/MS spectra of A_10_, which was 16 Da more than that of A_8_ at *m*/*z* 455, and also formed via loss of a HCOOH group (46 Da) from the parent ion (Figure 3). The MS^3^ spectra of A_10_ (*m*/*z* 517→471), which was similar to that of A_8_ (*m*/*z* 501→455), produced the characteristic fragment ions at *m*/*z* 453, 441 and 423, corresponding to the cleavages of a H_2_O (18 Da), a CH_2_O (30 Da), and a CH_2_O coupled with a H_2_O group (48 Da). Therefore, the A_10_ was tentatively identified to be the hydroxylated product of A_8_ or the dihydroxylated and dehydrogenated product of AA.

**Table 3:** Presupposed metabolic types and the *m*/*z* changes for potential metabolites of AA and MA.

Description	Molecular Formula Change	*m*/*z* Change
Decarboxylation	−CO_2_	−44
Hydroxymethylene loss	−CH_2_O	−30
Demethylation	−CH_2_	−14
Hydroxylation + dehydration	−2H	−2
Dehydrogenation	−2H	−2
Demethylation + hydroxylation	−CH_2_, +O	+2
Methylene to ketone	−2H+O	+14
Hydroxylation + dehydrogenation	−2H+O	+14
Methylation	+CH_2_	+14
Hydroxylation	+O	+16
Methyl to carboxylation	−2H+2O	+30
Hydroxylation and methylation	+CH_2_O	+30
Dihydroxylation+ dehydrogenation	+2O, −2H	+30
Dihydroxylation	+2O	+32
Tri-hydroxylation + dehydrogenation	+3O, −2H	+46
Tri-hydroxylation	+3O	+48
Glycine conjugation	+C_2_H_3_NO	+57
Sulfation	+SO_3_	+80
Hydroxylation and sulfation	+SO_4_	+96
Cysteine conjugation	+C_3_H_5_NOS	+103
Taurine conjugation	+C_2_H_5_NO_2_S	+107
S,N-Acetylcysteine onjugation	+C_5_H_7_NO_2_S	+145
Glucosidation	+C_6_H_10_O_5_	+162
Glucuronide conjugation	+C_6_H_8_O_6_	+176
Hydroxylation + glucuronide	+C_6_H_8_O_7_	+192
Glutathione conjugation	+C_10_H_15_N_3_O_6_S	+305
Glutathione conjugation	+C_10_H_17_N_3_O_6_S	+307

### 2.3. Identification of MA and Its Metabolites with Zebrafish Exposure

**Figure 4:**
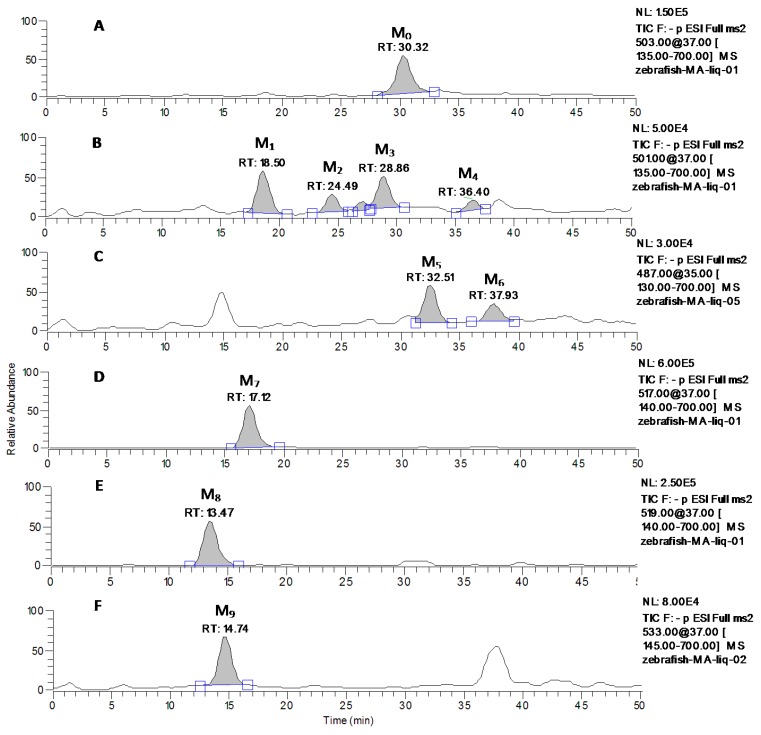
LC-MS/MS chromatograms of MA and its metabolites at *m*/*z* 503 (**A**), *m*/*z* 501 (**B**), *m*/*z* 487(**C**), *m*/*z* 517 (**D**), *m*/*z* 519 (**E**), *m*/*z* 533(**F**) with zebrafish exposure.

M_0_ was observed as the deprotonated molecule [M−H]^−^ at *m*/*z* 503 with retention time of 30.32 min (shown in Figure 4A). The retention time and MS^n^ spectra of M_0_ were the same as those of the standard compound, indicating that M_0_ was the unchanged parent drug MA.

**Figure 5:**
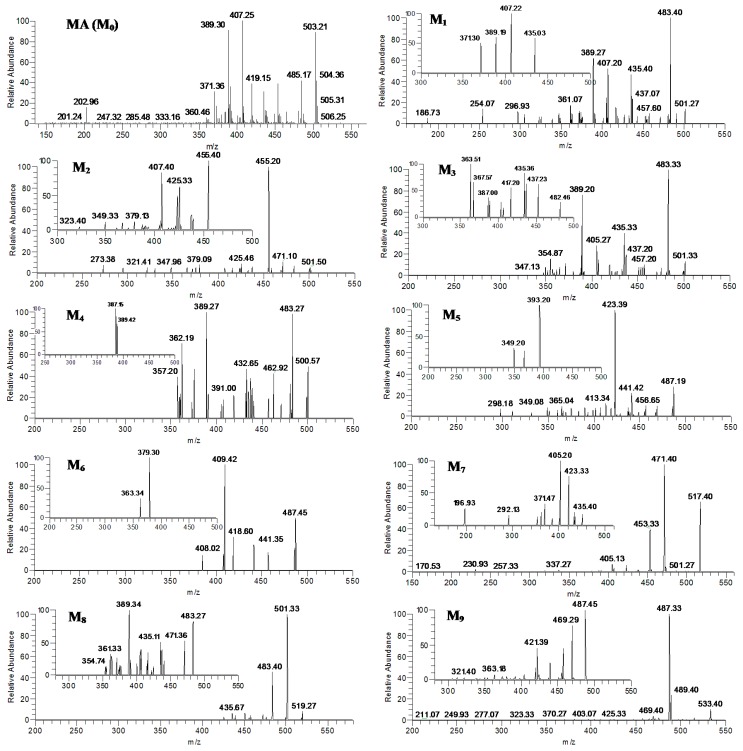
The MS^n^ (*n* = 2–3) spectra of the metabolites (M_0_–M_10_) of MA after zebrafish exposure: M_0_ (*m*/*z* 503); M_1_, M_3_ (*m*/*z* 501, *m*/*z* 501→483), M_2_ (*m*/*z* 501, *m*/*z* 501→455), M_4_ (*m*/*z* 501, *m*/*z* 501→389); M_5_ (*m*/*z* 487, *m*/*z* 487→423); M_6_ (*m*/*z* 487, *m*/*z* 487→409); M_7_ (*m*/*z* 517, *m*/*z* 517→471); M_8_ (*m*/*z* 519, *m*/*z* 519→483); and M_9_ (*m*/*z* 533, *m*/*z* 533→487).

The metabolite M_1_, M_2_, M_3_ and M_4_, eluted at 18.50, 24.49, 28.86 and 36.40 min, respectively, (seen in Figure 4B), showed the quasi-molecular ion at *m*/*z* 501 [M−H]^−^ that was 2 Da less than that of the parent MA (Figure 5). The MS/MS spectra of M_1_, M_3_ and M_4_ formed the common fragment ions at the *m*/*z* 483, which was also 2 Da than that of MA. The characteristic fragment ions at *m*/*z* 435, 407 and389, which were detected in the MS/MS spectra of M_1_, M_3_ and M_4_, were the same as that of MA, indicating that these metabolic reactions occurred on the functional groups outside structure. The characteristic fragment ion at *m*/*z* 455 was observed in the MS/MS spectra of M_2_, which was consistent with the neutral loss of a HCOOH (46 Da) from the quasi-molecular ion. The MS^3^ spectra of M_2_ (*m*/*z* 501→455), which was similar to that of A_8_ (*m*/*z* 501→455), formed the characteristic fragment ions at *m*/*z* 455, 439, 437, 425, 423 and 407. Based on the MS^n^ spectra data of M_1_, M_2_, M_3_ and M_4_ (shown in Table 2) and the fragmentation pathways of MA and AA (Figure 1), The metabolite M_1_, M_2_, M_3_ and M_4_ were identified as the isomers of dehydrogenation products of MA, and the metabolic pathway of M_2_ was different from that of M_1_ M_3_ and M_4_.

The MS/MS spectra of metabolite M_5_ and M_6_, eluted at 32.51 and 37.93 min, gave rise to the deprotonated ion [M−H]^−^ at *m*/*z* 487 that was 16 Da less than that of MA (seen in Figure 4C and Figure 5). The pseudo-molecular ion at *m*/*z* 487 of M_5_ exhibited the predominant product ions at *m*/*z* 441 and 423, indicating that there were neutral losses of a HCOOH (46 Da) and a HCOOH coupled with a H_2_O group (18 Da). The MS^3^ spectra of M_5_ (*m*/*z* 487→423) produced the major fragment ion at *m*/*z* 393 (see Figure 5), indicating that the neutral loss of a CH_2_O (30 Da) existed. The mass spectra of M_6_ were similar to that of AA, forming the characteristic fragment ions at *m*/*z* 441 and 409. Based on the MS^n^ spectra data of M_5_ and M_6_ (Table 2), the fragmentation pathways of MA and AA (Figure 1) and the data in Table 3, M_5_ and M_6_ was tentatively identified as the isomers of dehydroxylated product, or demethylation and dehydrogenation of MA. 

M_7_ was observed at 17.12 min (Figure 4D), which gave rise to the deprotonated ion [M−H]^−^ at *m*/*z* 517 (shown in Figure 5) and were 14 Da more than that of MA. The predominant deprotonated ions of M_7_ exhibited at *m*/*z* 471 and 453 (Figure 5 and Table 2), suggesting that there were sequential cleavages of a HCOOH (46 Da) and H_2_O group (18 Da). The MS^3^ spectra of M_7_ (*m*/*z* 517→471) produced the main fragment ions at *m*/*z* 453 ([m-H_2_O]^−^), 435 ([m-2H_2_O]^−^), 423 ([m-H_2_O-CH_2_O]^−^), 405 ([m-2H_2_O-CH_2_O]^−^) and 387 ([m-3H_2_O-CH_2_O]^−^) (see Figure 5 and Table 2) (here, m = M-HCOOH). The MS^4^ spectra of M_7_ (*m*/*z* 517→471→453) formed the main fragment ions at *m*/*z* 435 ([m-H_2_O]^−^), 425 ([m-CO]^−^), 423 ([m-CH_2_O]^−^), 407 ([m-H_2_O-CO]^−^), 405 ([m-H_2_O-CH_2_O]^−^) and 387 ([m-2H_2_O-CH_2_O]^−^) (here, m = M-HCOOH-H_2_O). The MS^4^ spectra of M_7_ (*m*/*z* 517→471→423) formed the main fragment ions at 405 ([m-H_2_O]^−^), 387 ([m-2H_2_O]^−^) and 357 ([m-2H_2_O-CO-2H]^−^) (here, m = M-HCOOH-H_2_O-CH_2_O). Therefore, M_7_ was identified as the hydroxylated and dehydrogenated product of MA. The data of MS^n^ spectra of M_7_ could further confirm this result. 

M_8_, which was eluted at 13.47 min, gave rise to the deprotonated quasi-molecular ion [M−H]^−^ at *m*/*z* 519 (Figure 4E). The major product ions at *m*/*z* 501 and 483 were observed in the MS/MS spectra of M_8_ (Figure 5), which was consisted with the sequential neutral losses of two H_2_O groups. The main product ions of the MS^3^ spectra of M_8_ (*m*/*z* 519→501) were similar to the MS/MS spectra of MA except less 2 Da (Figure 5 and Table 2). Based on the MS^n^ spectra data of M_8_ (*m*/*z* 519→501, 519→483, 519→501→483) (Table 2), the fragmentation pathways of MA (Figure 1) and the data in Table 3, M_8_ was identified as hydroxylated product of MA.

The metabolite M_9_, eluted at 14.74 min (shown in Figure 4F), gave rise to the quasi-molecular ion [M−H]^−^ at *m*/*z* 533, which were 14 Da more than that of M_8_ and 30 Da more than that of MA. The characteristic product ions from the MS/MS spectra of M_9_ exhibited at *m*/*z* 489 and 487 (see Figure 5), suggesting that there were the cleavages of a CO_2_ (44 Da) and a HCOOH group (46 Da). The MS^3^ spectra of M_9_ (*m*/*z* 533→487) produced the main fragment ions at *m*/*z* 469 ([m-H_2_O]^−^), 457 ([m-CH_2_O]^−^), 439 ([m-H_2_O-CH_2_O]^−^) and 421 ([m-2H_2_O-CH_2_O]^−^) (here, m = M-H-HCOOH). Based on the MS^n^ spectra data of M_9_ (see Table 2), the fragmentation pathways of MA and the data in Table 3, M_9_ was tentatively elucidated as the hydroxylated and dehydrogenation product of M_8_ or the dihydroxylated and dehydrogenated product of MA. 

### 2.4. Verification of Metabolites by Authentic Standards

To further confirm the metabolite identifications, two major metabolites A_7_ and M_6_ that were proposed as the hydroxylated product of AA and the dehydroxylated product of MA, respectively, were verified by using the authentic standards. Comparative analysis of the fragment ions from the MS^n^ spectra and retention times between the metabolites and authentic standards showed that the metabolite A_7_ well matched with the authentic standard of MA and M_6_ is in almost accordance with that of AA (shown in Figure 2A, Figure 3, Figure 4A, Figure 5 and Table 2), further supporting the original structure characterizations.

### 2.5. Discussion

Zebrafish is excellent model animal and widely used in drug screening and toxicological studies owing to its many advantages such as high-throughput, low cost and high efficiency, *etc.* [17,28,29]. Many results indicated that the developmental, biochemical and physiological processes were highly conserved between zebrafish and mammals, and drugs interacted with the biomacromolecules in zebrafish usually produced the similar pharmacological effects in human and mammals [18] Especially, the metabolic enzyme cytochrome P_450_ family was markedly expressed in zebrafish [19] that were very suitable for studying phase I drug metabolites. Our investigation confirmed that the zebrafish model could imitate the regular methods in elucidating the phase I metabolites and metabolic mechanism, which provided the further useful evidence for the possibility and reasonability using adult zebrafish in the drug metabolism study.

The hyphenated MS techniques are frequently the initial choice for drug metabolite detection and identification because of their sensitivity and convenience. It confirmed that the mass fragmentation patterns of metabolites are frequently similar to that of the parent compounds, thus, the analysis of fragmentation pattern of parent compound is very important and helpful for the metabolite characterizations [30] Actually, the mass spectral patterns of the parent drugs could serve as the templates in elucidation of the structures of the proposed metabolites of AA and its analogue MA. Determination of the metabolite structure was facilitated by the fact that the parent compound undergoes extensive and well definable fragmentation under the MS-MS conditions. Based on the above consideration, we analyzed and illustrated the detail fragmentation pathways of AA and its analogue MA in the first step of our work.

The most frequently applied strategy for the identification of unknown metabolites in biological matrix was as follows: firstly, scan of the product ions from the parent drug, and then, scan of the precursor ions and/or neutral loss of the biological samples, finally, scan of the product ions for the suspected or predicted metabolites. The metabolites of drug could be identified based on these experiment data and being aware of the typical metabolic pathways [31,32,33]. In this investigation, we firstly developed a novel method for rapid identification of the main metabolites in biological samples treated with AA or MA, which was the predictive multiple reactions monitoring (MRM) and targeted accurate mass measurement. The predictive MRM method was generated on the basis of the expected mass difference, the known fragmentation pathways and the *in vivo* metabolic rules of AA and its analogues (shown in Table 3) [34,35,36,37,38,39,40,41]. Then, an efficient LC/IT-MS/MS method with MRM mode was developed for the rapid screening and identification of the *in vivo* metabolites of AA and MA. In targeted accurate mass screening, the accurate mass of a suspected metabolite is calculated via the known metabolic pathways and the expected *m*/*z* changes. The MS^n^ spectra of the potential metabolites were obtained via fragmentation of the deprotonated molecules and used for more precise structural identification.

**Figure 6:**
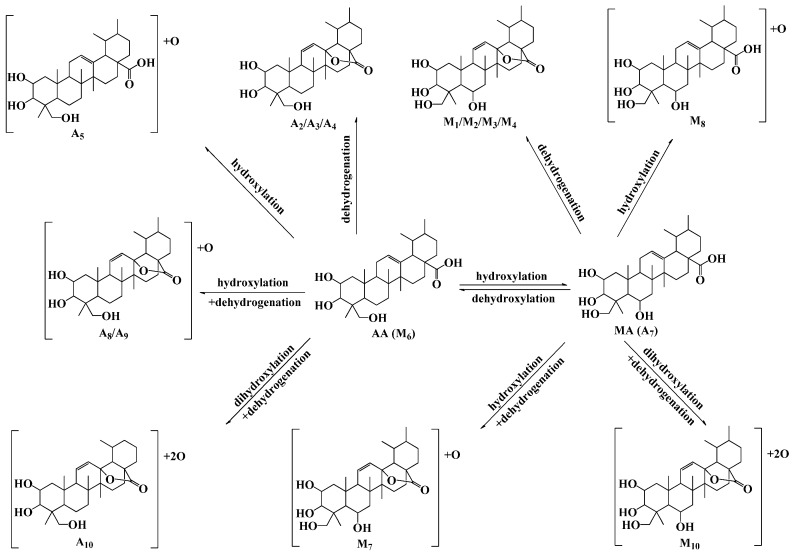
The main putative metabolites and proposed metabolic pathways of AA and MA in zebrafish.

Based the above methods, ten phase I metabolites of AA (A_1_–A_10_) and nine phase I metabolites of MA (M_1_–M_9_) were observed and identified in the solution after zebrafish was exposed to the compound. AA and MA were bio-transformed to phase I metabolites in the similar metabolic pathways, which formed from dehydrogenation, hydroxylation, hydroxylation and dehydrogenation, methylene to ketone reaction, dihydroxylation and dehydrogenation, demethylation and dehydrogenation, and dehydroxylation reaction. The proposed structures and metabolic pathways of these metabolites were also elucidated and analyzed (shown in Figure 6), which were coincident with the known metabolic rules of AA and its analogues.

## 3. Experimental Section

### 3.1. Chemicals and Reagents

Asiatic acid (AA) and mdecassic acid (MA) were obtained from Chengdu Must Biotechnology Co., Ltd (Chengdu, Sichuan, China). The purities of AA and MA were both more than 99%. Methanol and acetonitrile were purchased from Fisher Scientific (Fair lawn, NJ, USA) as the HPLC grade. Formic acid was purchased from Dikma Reagent Company (Beijing, China) as the HPLC grade. Triply distilled water was used in sample analysis. All other chemicals, reagents and solvents used were of analytical grade.

### 3.2. Apparatus and Analytical Conditions

The LC/IT-MS^n^ system, controlled by the Xcalibur^®^ (version 1.3) software, was consisted of a HPLC system (Series 1100, Agilent technology, Palo Alto, CA, USA) including a G1312A binary pump, a G1379A vacuum degasser and G1313A autosampler. The HPLC system was coupled to the Finngan LCQ Deca XP ion-trap spectrometer equipped with electrospray source (Thermo Finnigan, San Jose, CA, USA). Separation and determination of the analytes were achieved on a Symmetry^®^ C_18_ column (150 mm × 2.1 mm, i.d., 5 μm; Waters, Ireland) at ambient temperature. The mobile phase was composed of acetonitrile (A) and water with 0.05% formic acid (B), which was pumped at a flow-rate of 0.2 mL/min. The gradient program was performed in the following manner: 28% A at 0–2 min, 35% A at 18 min, and 50% A at 38–50 min. The sample injection volume was 10 μL with a run time of 50 min for each sample. Following a period of optimization, the ESI-MS^n^ was operated at the sheath flow rate of 40 psi with an ion spray voltage of 4.5 kV, and a heated capillary temperature of 350 °C. The MS^n^ product ion spectra were produced by collision induced dissociation (CID) of the deprotonated molecule ion [M−H]^−^ of the analyte at the respective HPLC retention time with the isolation width (the *m*/*z*) of 1.0. The collision energy for the analyte was in the range of 25%–38%, depending on the structures of different compounds.

### 3.3. Animal Protocol

The wild type (TU strain) zebrafish (D. rerio, mixed sex, 6 months in age, 5 cm in length, 0.25 g in mass) were obtained from the Laboratory Animals Center of Capital Medical University (LACCMU, Beijing, China). All studies on animals were in accordance with the guidelines of the Committee on the Care and Use of Laboratory Animals in China (CCULA, Beijing, China). The animal experiments welfare committee of Capital Medical University (CMU, Beijing, China) had approved the protocols of the animal studies.

### 3.4. Metabolism of AA and MA in Adult Zebrafish 

Prior to the experiments the zebrafish were acclimated under 14:10 h light-dark cycle and constant temperature (25 °C ± 2 °C) for at least two weeks in 10 L tanks equipped with a re-circulating fresh water in zerafish feeding system (Far East Instrument Co., Ltd., Taiwan, China) with air pump, biological and mechanical filtration system and ultraviolet lamps. The physicochemical properties of water such as the dissolved oxygen, pH, temperature, and salinity were monitored daily. When the zebrafish were exposed to the drug solution, drug metabolism consequently occurred, and the metabolites were continuously produced to delivery into the solution [42]. Comparative analysis of the component change of solution between the experiment and control groups could provide significant information about drug metabolism.

The experiments were performed in thermostat waterbath at 25 °C. The concentrations of the test compounds were determined and did not influence the activity of zebrafish for at least 48 h. Thirty zebrafish were randomly assigned into three groups and each group had ten zebrafish. After fasting for 12 h, ten zebrafish of each test group were exposed in 300 mL of water with AA (15 μg/mL) and MA (15 μg/mL), respectively. The control group was exposed in 300 mL of the water without these compounds. The samples were collected from the solution contained the test drug after the zebrafish had been exposed to the test drug for 36 h. All the samples were stored at liquid nitrogen prior to their analysis.

### 3.5. Sample Preparation

The solution sample (20 mL) of each group was freeze-dried to dryness, and the residue was dissolved in 0.5 mL methanol and vortexed for 2 min. After centrifugation at 14,000 rpm/min for 10 min, each supernatant was filtered with a 0.22 μm of membrane and an aliquot of 10 μL were directly injected into the LC-MS system for metabolite analysis.

## 4. Conclusions

This study firstly described the application of LC/IT-MS^n^ method using negative ion mode and collision induced dissociation to acquire the fragmentation pathways of AA and its analogue MA, and elucidate their *in vivo* metabolites. The main characteristic fragment ions of AA and MA were formed via cleavage of alicyclic ring, keto-enol tautomerism and retro-Diels-Alder cleavage on the C ring from their structures. Ten phase I metabolites of AA and nine phase I metabolites of MA were observed and identified in the solution after zebrafish were exposed to the drug.

This investigation confirmed that zebrafish model could imitate the regular methods in elucidating the drug phase I metabolism, which provided further useful evidence for the possibility and reasonability using adult zebrafish in the drug metabolism. With the advantages of lower cost and higher efficiency, zebrafish could be used as an effective metabolic model in drug metabolism research. Our investigation would also contribute to obtain the novel information on the structural elucidation, *in vitro* fragmentation of mass spectra, *in vivo* metabolites, intermediate process and metabolic mechanism of pentacyclic triterpenoids, which would be helpful to better understand the safety and efficacy of these compounds.
